# Clinical Course and Treatment of Patients With Apical Aneurysms Due to Hypertrophic Cardiomyopathy

**DOI:** 10.1016/j.jacadv.2024.101195

**Published:** 2024-08-27

**Authors:** Mark V. Sherrid, Daniele Massera, Samuel Bernard, Nidhi Tripathi, Yash Patel, Vivek Modi, Leon Axel, Soheila Talebi, Muhamed Saric, Elizabeth Adlestein, Isabel Castro Alvarez, Maria C. Reuter, Woon Y. Wu, Yuhe Xia, Brian B. Ghoshhajra, Danita Y. Sanborn, Michael A. Fifer, Daniel G. Swistel, Bette Kim

**Affiliations:** a Hypertrophic Cardiomyopathy Program; Leon Charney Division of Cardiology, Department of Medicine, NYU Grossman School of Medicine, New York City, New York, USA; b Echocardiography Laboratory, Leon Charney Division of Cardiology; Department of Medicine, NYU Grossman School of Medicine, New York City, New York, USA; c Division of Cardiology, Mount Sinai West and Mount Sinai Morningside, New York City, New York, USA; d Department of Radiology, NYU Grossman School of Medicine, New York City, New York, USA; e Division of Biostatistics, NYU Grossman School of Medicine, New York City, New York, USA; f Department of Radiology, Massachusetts General Hospital, Boston, Massachusetts, USA; g Echocardiography Laboratory, Massachusetts General Hospital, Corrigan Minehan Heart Center, Boston, Massachusetts, USA; h Hypertrophic Cardiomyopathy Program, Corrigan Minehan Heart Center, Massachusetts General Hospital, Boston, Massachusetts, USA; i Department of Cardiothoracic Surgery, NYU Grossman School of Medicine, New York City, New York, USA; j Cardiomyopathy Program, Mount Sinai West and Mount Sinai Morningside, New York City, New York, USA; k Echocardiography Laboratory, Mount Sinai West and Mount Sinai Morningside, New York City, New York, USA

**Keywords:** apical hypertrophic cardiomyopathy, apical left ventricular aneurysm, hypertrophic cardiomyopathy, left ventricular aneurysm, sudden death, ventricular arrhythmias

## Abstract

**Background:**

There is controversy about risk of malignant arrhythmias and stroke in patients with apical aneurysms in hypertrophic cardiomyopathy (HCM).

**Objectives:**

The aim of this study was to estimate the associations of aneurysm size and major HCM risk factors with the incidence of lethal and potentially lethal arrhythmias and to estimate incidence of unexplained stroke.

**Methods:**

In 108 patients (age 57.4 ± 13.5 years, 37% female) from 3 HCM centers, we assessed American Heart Association/American College of Cardiology guidelines risk factors and initial aneurysm size by echocardiography and cardiac magnetic resonance imaging and assessed outcomes after median 5.9 (IQR: 3.7-10.0) years.

**Results:**

Implantable cardioverter defibrillator discharges or sudden cardiac death (SCD) occurred in 21 (19.4%) patients. Of patients with a risk factor, 55% subsequently had ventricular tachycardia (VT), ventricular fibrillation (VF), or SCD at follow-up, compared with 10% in those who did not (*P* < 0.001). The upper tercile of size had a 5-year cumulative risk of 35%, while the lower tercile had 5-year risk of 6% (*P* = 0.0046). In those with the smallest aneurysms <2 cm^2^ and also without risk factors VT, VF, or SCD occurred in only 2.5%. Clinical atrial fibrillation (AF) was prevalent, occurring in 49 (45%). Stroke was commonly associated with AF. Stroke without conventional cause had an incidence of 0.5%/year. Surgery in 19% was effective in reducing symptoms. VT ablation and surgery were moderately effective in preventing recurrent VT.

**Conclusions:**

Risk factors and aneurysm size were associated with subsequent VT, VF, or SCD. Patients with aneurysms in the lowest tercile of size have a low cumulative 5-year risk. Clinical AF occurred frequently. Stroke prevalence in absence of known stroke etiologies is uncommon and comparable to risk of severe bleeding.

Left ventricular (LV) apical aneurysms occur in 3 to 5% of patients with hypertrophic cardiomyopathy and are associated with lethal or potentially lethal ventricular arrhythmias and heart failure symptoms.^1^, ^2^, ^3^, ^4^ Rowin reported a 4.7%/year incidence of appropriate implanted cardioverter defibrillator (ICD) discharges or sudden cardiac death (SCD). Lee showed a relatively high frequency as well, albeit with a lower event rate, 1.8%/year. Aneurysms are found in varying sizes, from quite small, barely detectable with noncontrast echocardiography, to quite large, up to ∼20 ml.^5^ The continuous distribution of aneurysm size (rather than a bimodal distribution) and their very high association with mid-LV or apical obstruction indicates that small aneurysms, previously called pouches, and larger aneurysms are not 2 distinct entities but rather variations of the same pathology.^5^ Small aneurysms are decidedly more common. The question arises whether they should all be treated the same.^4^ American guidelines list presence of an aneurysm as a high-risk feature for consideration of ICD implantation regardless of size. European Society of Cardiology (ESC) guidelines do not include apical aneurysms as a SCD risk factor. Investigators have found that smaller aneurysms convey less risk.^4^ In the present investigation, we analyze whether hypertrophic cardiomyopathy (HCM) risk factors and aneurysm size are associated with a higher incidence of ventricular tachycardia (VT), ventricular fibrillation (VF), and SCD. We tabulate the frequency of AF and also analyze incidence of unexplained strokes that might, or might not, be associated with aneurysms. We report on the symptomatic response to palliative surgery performed for resistant symptoms.

## Methods

This was a retrospective study of consecutive patients diagnosed with apical aneurysms of varying size from 3 tertiary-referral HCM programs: NYU Langone Health, Massachusetts General Hospital, and Mount Sinai West (formerly Roosevelt Hospital) from 2000 to 2019. Institutional review board approval was obtained from participating centers. We previously reported echo and cardiac magnetic resonance imaging (CMR) exams performed at initial consultation, characteristics of these patients, including diagnostic criteria for HCM, and how consecutive patients were identified for inclusion and exclusion.^5^ Aneurysms were defined as dilation of the LV apex with relative thinning of the walls and severe hypokinesia, akinesia, or dyskinesia of affected segments. From chart review, we ascertained the presence of clinical SCD-related American Heart Association/American College of Cardiology guidelines (AHA/ACC) major (2a) risk factors: 1) sudden death attributable to HCM in ≥1 first-degree or close relatives who are ≤50 years of age; 2) massive left ventricular hypertrophy ≥30 mm in any LV segment; 3) ≥1 recent episodes of syncope suspected by clinical history to be arrhythmic; and 4) LV systolic dysfunction (LV ejection fraction [LVEF] <50%). ESC 5-year risk scores were calculated for each patient.^6^ Clinical follow-up and survival were obtained from chart reviews and telephone. Lethal or potentially lethal arrhythmias were defined as VT or VF requiring device therapy or SCD. VT storm was defined as 3 or more episodes of sustained VT in a 24-hour period requiring therapy. ICD interventions were analyzed by local centers’ electrophysiologists with categorization of appropriate discharges as VT or VF. Atrial fibrillation was tabulated if a symptomatic clinical event occurred diagnosed on an office visit, emergency room, or hospital admission. Diagnosis of stroke, central nervous system infarction, was based on modern criteria requiring evidence of symptomatic cerebral or retinal focal ischemic injury with symptoms and signs persisting ≥24 hours or until death, or by imaging evidence of central nervous system focal ischemic injury.^7^ Transient ischemic events (TIAs) were not tabulated as stroke unless there was imaging evidence of infarction. LV apical thrombus was not considered equivalent to stroke. Major bleeding was as defined by the International Society on Thrombosis and Haemostasis (Supplemental Appendix).

### Echocardiography and CMR measurements

A detailed description of methods and measurements made by 2D and Doppler echocardiography and CMR in these patients has been reported previously ^5^ (Supplemental Appendix). We measured echo area and volume of the aneurysm in 2 orthogonal apical views, tabulating the average. CMR aneurysm area was measured in 2 orthogonal long-axis views, tabulating the average. Late gadolinium enhancement (LGE) images were again independently reviewed, and qualitative extent of LGE was categorized as either isolated to the apical cap or extending toward the level of obstruction and PMs.

### Surgery

Patients were referred for surgery if they had medication-resistant New York Heart Association class (NYHA)-functional class III symptoms or, less commonly, for relief of recurrent VT.^8^ The large majority of patients were operated on by 1 surgeon (D.G.S.). Surgery tailored to individual pathology was only performed in mid-LV obstruction patients, which comprise 95% of aneurysm cases.^3^^,^^5^ In patients with focal, limited-length mid-LV obstruction, small aneurysms, and compliant myocardium, myectomy was performed through the aortotomy and extended to the apex under direct visualization. When appropriate, papillary muscle (PM) thinning was added as an ancillary procedure.^5^^,^^8^ The decision to open the apex with a ventriculotomy was based on presence of both a large aneurysm and a long longitudinal extent of mid-LV obstruction (>1.5 cm). Myectomy and PM thinning were performed from this exposure. If both exposures were used, myectomies were connected. The decision to perform aneurysmectomy was based on size of the aneurysm, extent of preoperative LGE, and direct surgical inspection of degree of gross scar. Survival after surgery and change in NYHA functional class were assessed. Frequency of appropriate ICD interventions for ventricular arrhythmias was tabulated before and after surgery and VT ablation procedures. Results afterward were categorized as no further ICD discharges for VT, decreased, or ineffective.

### Statistics

Continuous and normally distributed variables are reported as mean ± SD; data with skewed distribution are presented as median (IQR). Categorical variables are presented as number and percentage. Differences between prevalence of events in patients with/without risk factors and in aneurysm size between patients with/without VT, VF, and SCD were assessed using Fisher’s exact or chi-square test for categorical variables and Student’s t or Mann-Whitney U test for continuous variables, respectively. We analyzed outcome of interest, time to ICD discharge for VT/VF, or sudden death utilizing the Fine-Gray model to estimate HRs for various predictors while accounting for competing risk of death from other causes. Covariates included in analysis were selected based on clinical relevance and likelihood to influence ICD discharge or sudden death. Area of aneurysm, LVEF, and maximum LV wall thickness were log-transformed to reduce impact of large values. Survival curves were estimated using Kaplan-Meier method, and differences among terciles of biplane echo aneurysm area for occurrence of VT, VF, or SCD were determined using log-rank tests. Analyses were performed in R version 4.2.3.

## Results

**Figure 1:**
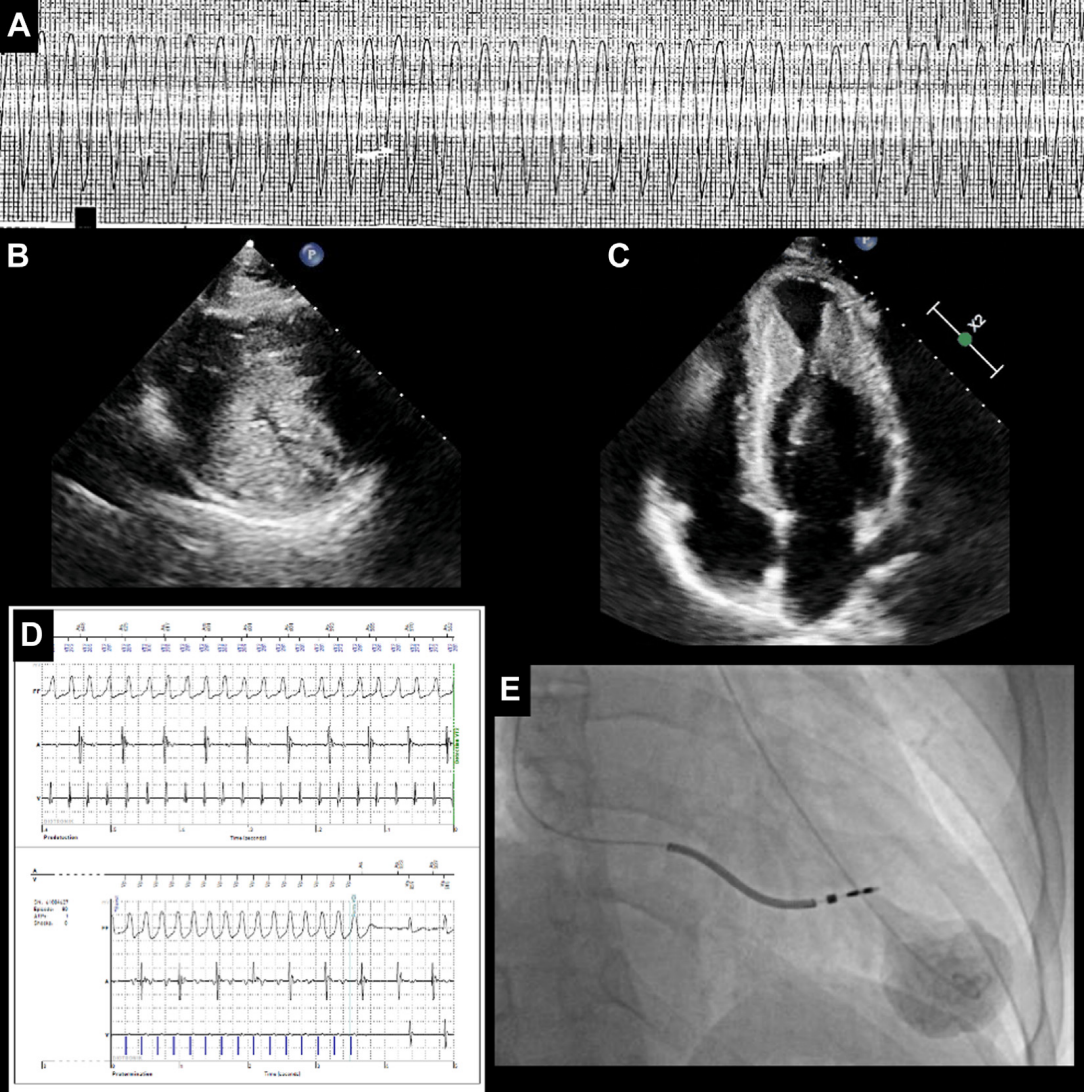
**Large HCM Apical Aneurysm** A 21-year-old with (A) rapid VT on admission; (B) midsystolic complete emptying of the mid-LV; (C) early systolic complete emptying and the akinetic aneurysm; (D) ATP termination of 1 of very many monomorphic VT events; (E) apical aneurysm with complete mid-LV systolic emptying and obstruction. ATP = antitachycardia pacing; HCM = hypertrophic cardiomyopathy; LV = left ventricular; VT = ventricular tachycardia.

**Figure 2:**
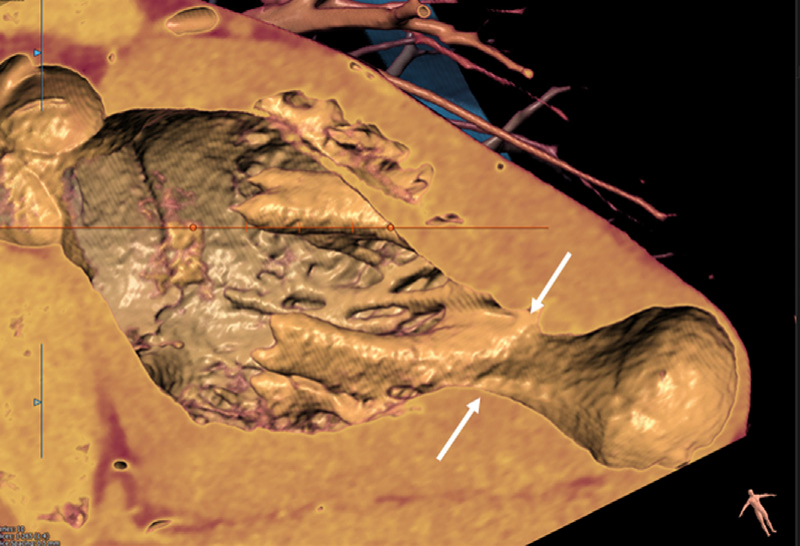
**Rendered Tomographic CT Scan** The mid-LV walls contract circumferentially around a hypertrophied PM at its insertion into the LV wall (white arrows). The PM acts as a contributing central plug ^5^^,^^8^ Courtesy of Alan Vainrib, MD. CT = computed tomography; LV = left ventricular; PM = papillary muscle.

There were 108 patients, whose average age was 57.4 ± 13.5 years, and 40 (37%) were female (see Table 1). Racial distribution is discussed in the Supplemental Appendix. Follow-up duration was 5.9 [3.7-10.0] years after initial consultation. Graphics of a representative patient with a large apical aneurysm are shown in Figures 1 and 2 and Video 1. Average NYHA functional class was 2.0 ± 0.8; 36 patients (33%) had class ≥III symptoms.

**Table 1:** Initial Clinical Characteristics of 108 HCM Patients With Apical Aneurysms

Age (y)	57.4 ± 13.5
Female	40 (37%)
Race	
White	63 (58.3)
Black	27 (25)
Asian	14 (13)
Hispanic	4 (3.7)
NYHA functional class	2.0 ± 0.8
History of clinical atrial fibrillation	25 (23)
Prior stroke	4 (4)
Peripheral emboli	0
Reason for anticoagulation	
AC for AF	27 (25)
AC for apical thrombus	5 (5)
AC for AF and thrombus	1 (1)
AC for pulmonary emboli	1 (1)
Anticoagulation	34 (31)
Warfarin	16 (15)
NOAC	18 (17)
Epicardial CAD >70% stenosis	0
Symptomatic VT	16 (15)
ICD before initial visit	9 (8)
Appropriate discharge before initial visit	4 (4)
Prior sustained VT or cardiac arrest	7 (6.4)
Major AHA/ACC risk factor	20 (18.5)

Values are mean ± SD or n (%).

AC = anticoagulation; AF = atrial fibrillation; AHA/ACC = American Heart Association/American College of Cardiology guidelines; CAD = coronary artery disease; HCM = hypertrophic cardiomyopathy; ICD = implanted cardioverter defibrillator; NOAC = novel anticoagulant; NYHA = New York Heart Association class; VT = ventricular tachycardia.

### Ventricular tachycardia, fibrillation, and sudden death

A total of 61 patients (56%) had an ICD implanted by follow-up (including 9 before their initial visit). Twenty-one (19.4%) of 108 patients had an appropriate discharge or SCD. Age at time of events was 55.8 ± 16 years including 6 patients <45 years of age. Eight (38%) with appropriate discharges or SCD were >60 years of age. Seven patients had sustained VT or cardiac arrest before their initial visit; of these, 5 had recurrent VT with appropriate discharge by follow-up. Twenty patients had appropriate discharges after their initial visits, 33% of patients with implants. Two of the discharges were for VF; thus, the overwhelming majority were antitachycardia pacing (ATP) for VT (90%). There were >1 discharges in 16 patients (15%); >5 discharges in 9 (8%); and VT storm occurred in 6 (5.5% of the whole cohort and 33% of those with VT). VF occurred in 1 patient after a prolonged bout of AF; 2 episodes of VF occurred in a second patient who was devolving to severe LV systolic dysfunction. One patient, aged 83, who did not have an ICD was unexpectedly found dead at home and tabulated as SCD. Table 2 shows characteristics of patients with ICD discharges and SCD and the complexity of their care. There was no difference in age of initial evaluation in patients with and without lethal or potentially lethal events (53.2 ± 16 vs 58.4 ± 13, *P* = 0.11).

**Table 2:** ICD Discharges or SCD in 21 Patients

Age at First ICD Discharge or SCD, y	# Days With DCs	# of DCs	VT Storm	AAD	VT Ablate, # of Procedures	No Further DC for VT (NoDC) or Ineffective (I)	Surgery for VT	No Further DC for VT (NoDC), Decreased DCs (D) or Ineffective (I)	Outcome and Comment
21.5	4	6	Y	A, S			Y	NoDC	No need for VT ICD therapy after surgery
30.9	1	2			Y, 2	NoDC			No need for VT ICD therapy after ablation
38.6	1	9	Y						ATP
39.0	>3	4		A					VF after AF x 3. VF without AF Transformation LV dysfunction. Transplant
40.9	1	2			Y, 1	NoDC			No need for VT ICD therapy after ablation
44.5	1	1		Di			Y	NoDC	No need for VT ICD therapy after surgery
45.8	8	>75		M, A	Y, 4	I			VT controlled after multiple ablations and low dose amiodarone
49.8	3	5		Di, M					ATP
54.0	2	2					Y	NoDC	No need for VT ICD therapy after surgery
56.9	>20	>20					Y	D	Surgery eventually abolished VT
58.1	1	multiple	Y	S	Y, 1	NoDC			No need for VT ICD therapy after ablation
58.4	2	2		A, M, Di, P			Y	NoDC	No need for VT ICD therapy after surgery
59.0	1	4							ATP
63.4	3	9	Y	A, M			Y	I	Surgery, amio and mex abolished VT but recurred when amio stopped
66.8	4	Multiple			Y, 1	I	Y	D	Surgery decreased VT and DC frequency
67.4	1	1		Di					VF after several hours of AF. No recurrence on disopyramide
71.0	9	114	Y	A, Di, P	Y, 3	I	Y, A	D	No VT after surgery for 3 y and then VT storm; subsequent 3rd ablation with amio abolished VT
71.9	1	1			Y, 1	NoDC			No need for VT ICD therapy after ablation
75.4	3	5	Y						ATP
75.8	>10	>10		A	Y, 1	I			Continues to have VT rx'd ATP
83.2	SCD	SCD							SCD

A = amiodarone; AAD = antiarrhythmic drugs; Ab = abolished; Ablate = ablation; ATP = antitachycardia pacing; D = decreased ICD discharges; DCs = discharges; Di = disopyramide; I = ineffective for VT; ICD = implanted cardioverter defibrillator; M = mexilitine; NoDC = no discharges for VT; P = procainamide; SCD = sudden cardiac death; VT = ventricular tachycardia.

### Risk factors in the VT, VF, and SCD at follow-up patients

**Figure 3:**
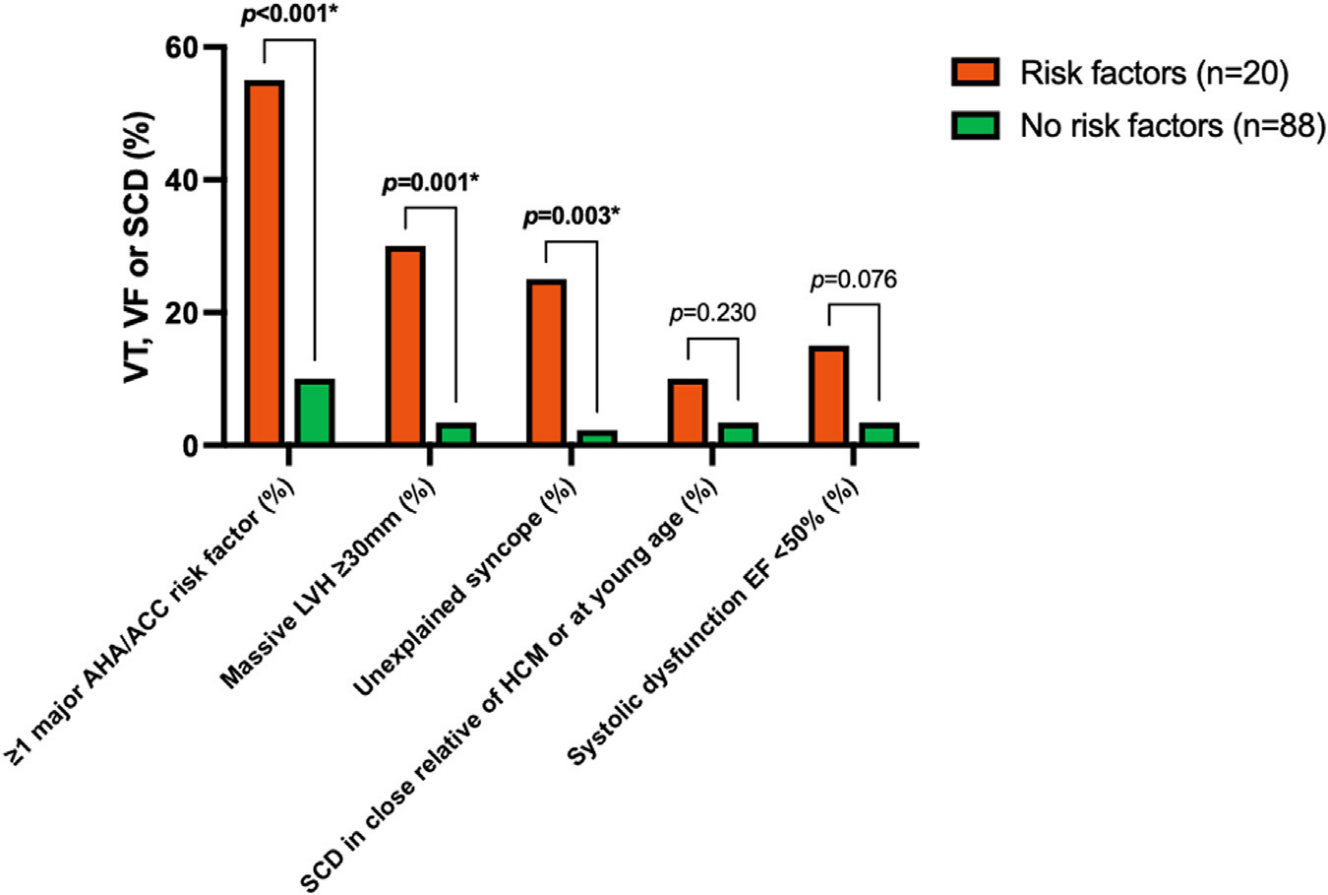
**Prevalence of Subsequent VT, VF, or SCD in Patients With a Major Risk Factor** Patients with risk factor (red) (n = 20) and those without (green) (n = 88). SCD = sudden cardiac death; VF = ventricular fibrillation; VT = ventricular tachycardia.

Of 108 patients, 20 patients had at least 1 major AHA/ACC risk factor other than aneurysm at initial visit, and 88 did not. Of 20 patients with a risk factor initially, 11 (55%) subsequently had VT, VF, or SCD compared with 88 without a risk factor, of whom 9 (10%) subsequently had VT, VF, or SCD (*P* < 0.001) (Table 3, Figure 3). Of 101 patients without prior sustained VT or cardiac arrest, 16 had ≥1 ACC/AHA major risk factor and 85 did not. Of 16 patients with a risk factor initially, 8 (50%) subsequently had VT, VF, or SCD compared with 85 without a risk factor of whom 8 (9%) subsequently had VT, VF, or SCD (*P* < 0.001). Thus, presence of a major risk factor at initial visit was a potent predictor of subsequent malignant arrhythmias. Five-year ESC risk score was higher in VT, VF, or SCD patients than those without 3.47 ± 2.0 vs 2.21 ± 1.4 (*P* < 0.003), as would be expected by the prevalence of risk factors. However, 5-year risk was only ≥6% in 16% of patients (=ICD should be considered) who subsequently sustained VT, VF, or SCD and was only ≥4% in 24% (=ICD may be considered).

**Table 3:** Prevalence at Follow-Up of VT, VF, or SCD in 20 Patients With Major Risk Factors at Presentation Compared With 88 Patients Without Risk Factors

	Risk Factors (n = 20)	No Risk Factors (n = 88)	*P* Value
≥1 major AHA/ACC risk factor present	11 (55)	9 (10)	<0.001
Massive LVH >30 mm	6 (30)	3 (3.4)	0.001
Unexplained syncope	5 (25.0)	2 (2.3)	0.003
FMHx of SCD in close relative of HCM or at young age	2 (10)	3 (3.4)	0.23
Systolic dysfunction LVEF <50%	3 (15)	3 (3.4)	0.076

Values are n (%).

AHA/ACC = American Heart Association/American College of Cardiology guidelines; FMHx = family history; HCM = hypertrophic cardiomyopathy; LVEF = left ventricular ejection fraction; LVH = left ventricular hypertrophy; SCD = sudden cardiac death; VF = ventricular fibrillation; VT = ventricular tachycardia.

### Imaging in VT, VF, and SCD patients

Ninety-four patients (87%) had echos of adequate quality for quantification of aneurysm size. CMR was performed on 82 (76%) patients, less frequently performed from 2000 to 2010. Details of imaging results have been reported previously.^5^ The vast majority of patients, 103 (95%), had obstruction with mid or apical complete emptying on transthoracic echocardiography confirmed on multiple orthogonal views and on CMR, while only 5 (5%) were relatively hypokinetic/nonobstructed in the mid-LV. Only patients with clear endocardial borders with or without contrast were traced for aneurysm area and volume. Median echocardiographic aneurysm volume was 2.5 (IQR: 1.2-5.5) ml, and echo aneurysm area was 2.58 (IQR: 1.7-4.3) cm^2^. Median CMR area was 2.5 (IQR: 1-4.9) cm^2^.

**Figure 4:**
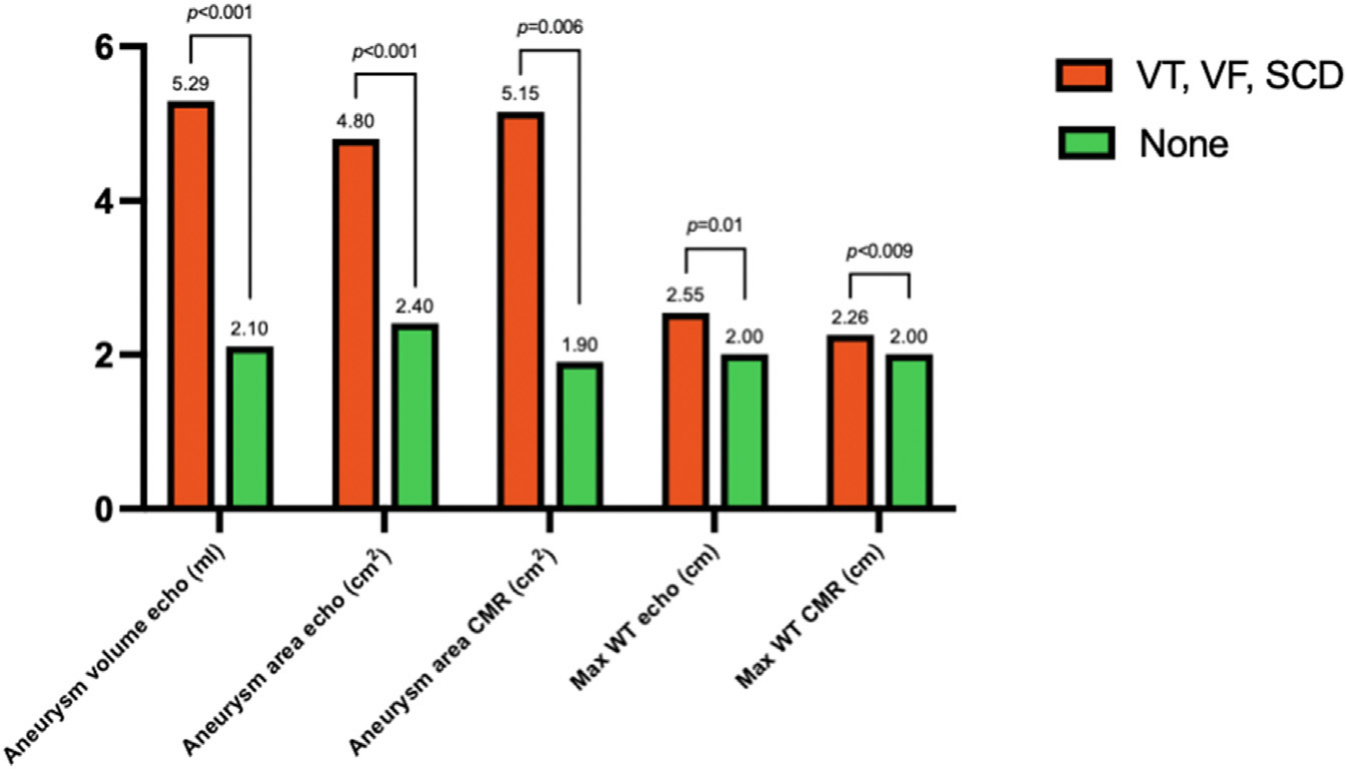
**Echo and CMR Measurements in Patients With and Without Subsequent VT, VF, or SCD** Patients with (n = 21) and without (n = 87) subsequent VT, VF, or SCD. CMR = cardiac magnetic resonance imaging; SCD = sudden cardiac death; VF = ventricular fibrillation; VT = ventricular tachycardia; WT = wall thickness.

**Figure 5:**
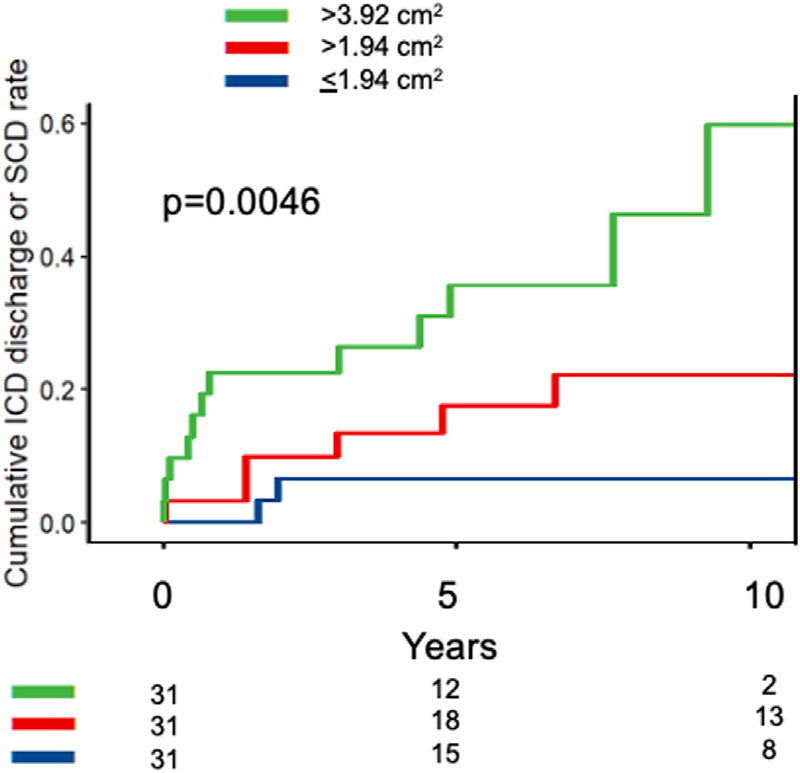
**Kaplan-Meier Event Rates for Subsequent VT, VF, or SCD by Tercile of Biplane Echo Aneurysm Area (cm**^**2**^**)** ICD = implanted cardioverter defibrillator; SCD = sudden cardiac death; VF = ventricular fibrillation; VT = ventricular tachycardia.

Echocardiographic aneurysm volume was greater in 21 patients who had sustained an appropriate discharge or SCD than in 87 who had not: 5.29 (IQR: 3.21-11.11) vs 2.10 (IQR: 0.88-4.10) ml, *P* < 0.001, as was 2D echo area: 4.80 (IQR: 2.64-6.43) vs 2.40 (IQR: 1.41-3.97) cm^2^, *P* < 0.001. CMR area was similarly larger 5.15 (3.00-8.10) vs 1.90 (IQR: 0.96-4.02) cm^2^, *P* = 0.006. Maximum LV wall thickness in mid-LV was greater in patients with appropriate discharges or SCD than those without, on echocardiography, 25.5 (20.0-30.2) vs 20.0 (18.0-24.0) mm, *P* = 0.01, and on CMR, 22.6 (22.0-28.0) vs 20.0 (17.3-23.0) mm, *P* < 0.009. Table 4 and Figure 4 shows echo and CMR measurements in patients with and without VT, VF, or SCD. Figure 5 shows incidence of VT, VF, or SCD over time from initial visit. The population was divided into terciles. With echo aneurysm area of >3.92 cm^2^ compared with >1.94 cm^2^ and ≤1.94 cm^2^, 5-year cumulative event rates were 35%, 17%, and 6%, respectively (*P* = 0.0046). In patients who had VT, VF, or SCD at follow-up but who had neither an ACC/AHA risk factor nor prior sustained VT or cardiac arrest initially, average area of their aneurysms was large, at 4.4 cm^2^.

**Table 4:** Comparison of 21 Patients With VT, VF, or SCD vs 87 Patients Without Malignant Ventricular Arrhythmia

	Appropriate ICD Discharge or SCD (n = 21)	No Appropriate ICD Discharge (n = 87)	*P* Value
Age at first visit, y	53.2 ± 16	58.4 ± 13	0.11
Male	14 (67)	55 (63)	0.97
Echo aneurysm volume, ml	5.29 (3.21-11.11)	2.10 (0.88-4.10)	<0.001
Echo aneurysm area, cm^2^	4.80 (2.64-6.43)	2.40 (1.41-3.97)	<0.001
CMR aneurysm area, cm^2^	5.15 (3.00-8.10)	1.90 (0.96-4.02)	0.006
Echo LVEF, %	65.00 (55.00-71.00)	70.50 (65.00-75.00)	0.007
Maximum echo wall thickness, mm	25.50 (20.00-30.20)	20.00 (18.00-24.00)	0.01
Maximum CMR wall thickness, mm	22.60 (22.00-28.00)	20.00 (17.25-22.95)	<0.009
Papillary muscle area, cm^2^	4.2 ± 3	3.2 ± 1	<0.03
% LGE	17.00 (6.00-25.00)	15.00 (7.00-22.50)	0.58
LGE located at cap only	2 (17)	28 (51)	
LGE extends to PMs	10 (83)	27 (49)	0.052

Values are mean ± SD, n (%), or median (IQR).

CMR = cardiac magnetic resonance imaging; ICD = implanted cardioverter defibrillator; IQR = interquartile range; LGE = late gadolinium enhancement; LVEF = left ventricular ejection fraction; PM = papillary muscle; SCD = sudden cardiac death; VF = ventricular fibrillation; VT = ventricular tachycardia.

There was no difference in percentage of late gadolinium enhancement (%LGE) in those with and without appropriate discharges or SCD (17.00 [IQR: 6.00-25.00] vs 15.00 [IQR: 7.00-22.50], *P* = 0.58). Only 6.7% of patients with LGE localized just at the apical cap had appropriate discharges or SCD, compared with 27.0% of patients with LGE extending up toward the level of obstruction (*P* = 0.052).

**Figure 6:**
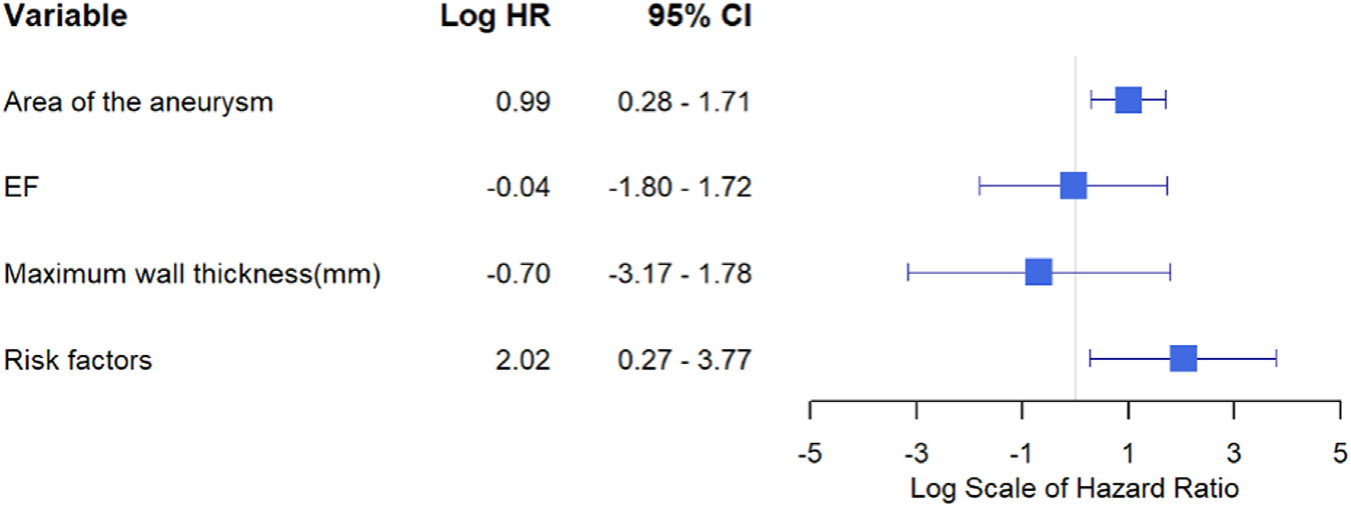
Multivariable Competing Risk Analysis Model for Predictors of Lethal or Potential Lethal Arrhythmia

**Figure 7:**
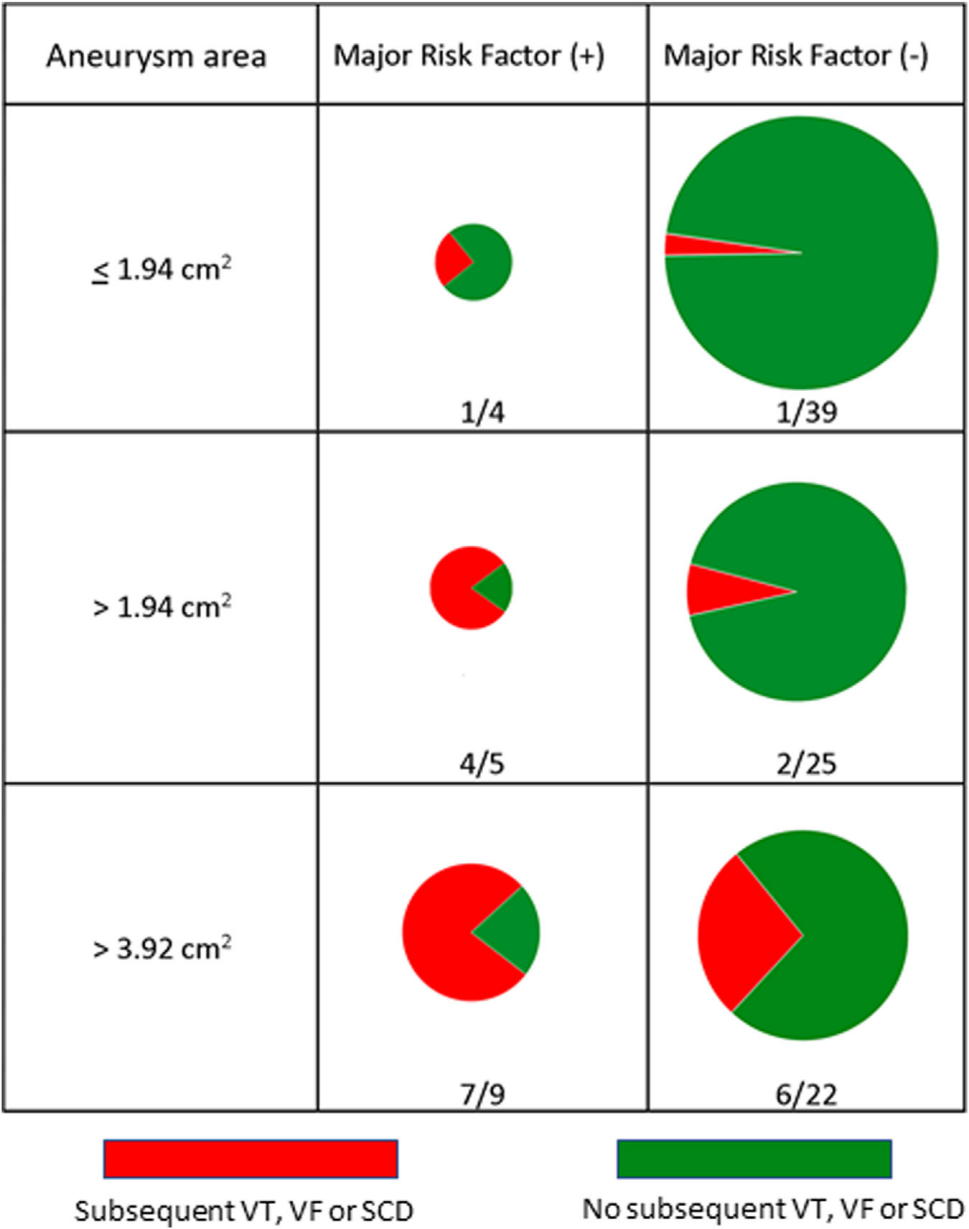
**Fraction of Patients With VT, VF, or SCD Stratified by Whether They Had a Major Risk Factor and Also by Aneurysm Echo Area** Only 1 of 39 patients (2.5%) had malignant arrhythmia who had no risk factor and a small aneurysm, compared with 7 of 9 (78%) who had both a risk factor and a large aneurysm. SCD = sudden cardiac death; VF = ventricular fibrillation; VT = ventricular tachycardia.

Multivariable analysis including presence of a major AHA/ACC risk factor, area of the aneurysm, LVEF, and maximum LV wall thickness showed that presence of a risk factor (HR: 7.56 [95% CI: 1.32-43.42], *P* = 0.023), and echo aneurysm area (HR: 2.70 [95% CI: 1.32-5.50], *P* = 0.007), were associated with higher risk of subsequent occurrence of VT, VF, or SCD when adjusting for other variables (Figure 6). The percentages of patients with VT, VF or SCD stratified by whether they had a major risk factor and also by aneurysm echo area are shown in Figure 7. Only 1 of 39 patients (2.5%) who had no risk factor and a small aneurysm had malignant arrhythmia, compared with 7 of 9 (78%) who had both a risk factor and a large aneurysm.

### Deaths

Death occurred in 12 (11.1%) patients. Deaths were noncardiac in 7 (6.5%) patients and HCM-related in 5 (4.6%) patients. One had sudden death, 1 died after surgery, 2 from heart failure, and 1 a day after atrioventricular node ablation. One patient with a large aneurysm without mid-LV obstruction underwent cardiac transplantation for refractory heart failure symptoms, and another patient has stable LV dysfunction, LVEF = 40%.

### Atrial fibrillation

Clinical AF occurred in 25 (23%) patients before their first consultation; by follow-up, it had occurred in 49 patients (45.4%). Fifty-six patients (52%) had received sustained systemic anticoagulation for at least a portion of their follow-up. In 47 patients this was for AF; in addition, 5 patients were anticoagulated because of thrombus in the aneurysm, 2 patients for their aneurysm without thrombus, and 2 patients for pulmonary embolism.

### Stroke

Nine strokes (8.3%) occurred in patients while under our care, average incidence 1.4%/year. Of these, 5 occurred in patients with new-onset clinical AF who were not anticoagulated or during a hiatus in anticoagulation. One patient had a stroke deemed due to a severe internal carotid stenosis that was subsequently stented. Only 3 patients had strokes with no conventional explanation (overall 2.8% of 108 patients), incidence 0.5%/year.

Four patients (3.7%) had strokes before they entered our care. One occurred with AF not on anticoagulation, 1 was due to a vertebral dissection, and 2 occurred without any explanation. Overall, before initial visit and by follow-up strokes occurred in 13 patients (12%) with 6 attributed to AF and 2 due to large vessel extracranial disease. Five patients had strokes (4.6% of 108 patients) with no conventional cause found.

**Figure 8:**
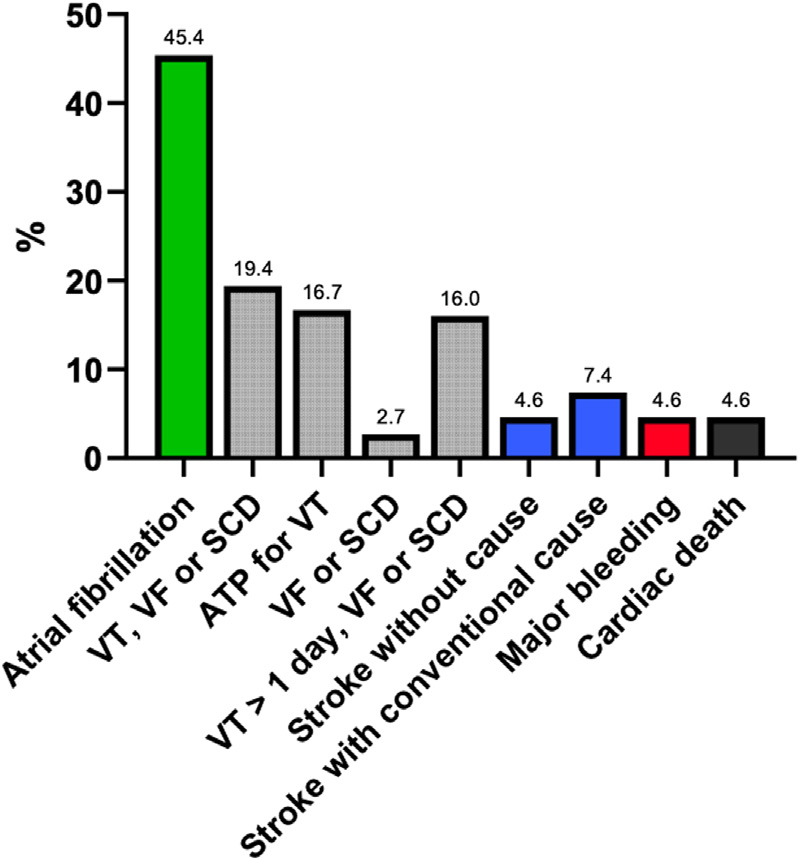
**Prevalence of Clinical Events in 108 HCM Aneurysm Patients Followed Median 5.9 Years** ATP = antitachycardia pacing; HCM = hypertrophic cardiomyopathy; SCD = sudden cardiac death; VF = ventricular fibrillation; VT = ventricular tachycardia.

Seven (6.5%) patients were found to have aneurysm thrombi; none of these patients had strokes. Five patients were subsequently given chronic anticoagulation. None of 13 patients who had strokes had aneurysm thrombi. Two patients had organized thrombi in the aneurysm at time of surgery; neither had experienced strokes. Major bleeding occurred under our care in 5 (4.6%) patients, 4 on anticoagulation (3 for AF, and 1 for pulmonary emboli). Prevalence of arrhythmias, stroke, and bleeding are shown in Figure 8.

### Pharmacotherapy for symptoms, antiarrhythmics, and VT ablation

All patients with limiting symptoms received a trial of beta-blocker, calcium channel blocker, or both. Amiodarone, sotalol, mexiletine, and disopyramide were the most frequently administered antiarrhythmics. Eight patients had VT ablations (44% of VT patients). Three patients had multiple ablations: 3 in 1 patient and 4 in another. Ablation was successful in abolishing VT in 4 patients but was ineffective in 4, requiring continued ATP and antiarrhythmic medication, and 2 required aneurysmectomy.

### Surgical myectomy and aneurysmectomy

**Figure 9:**
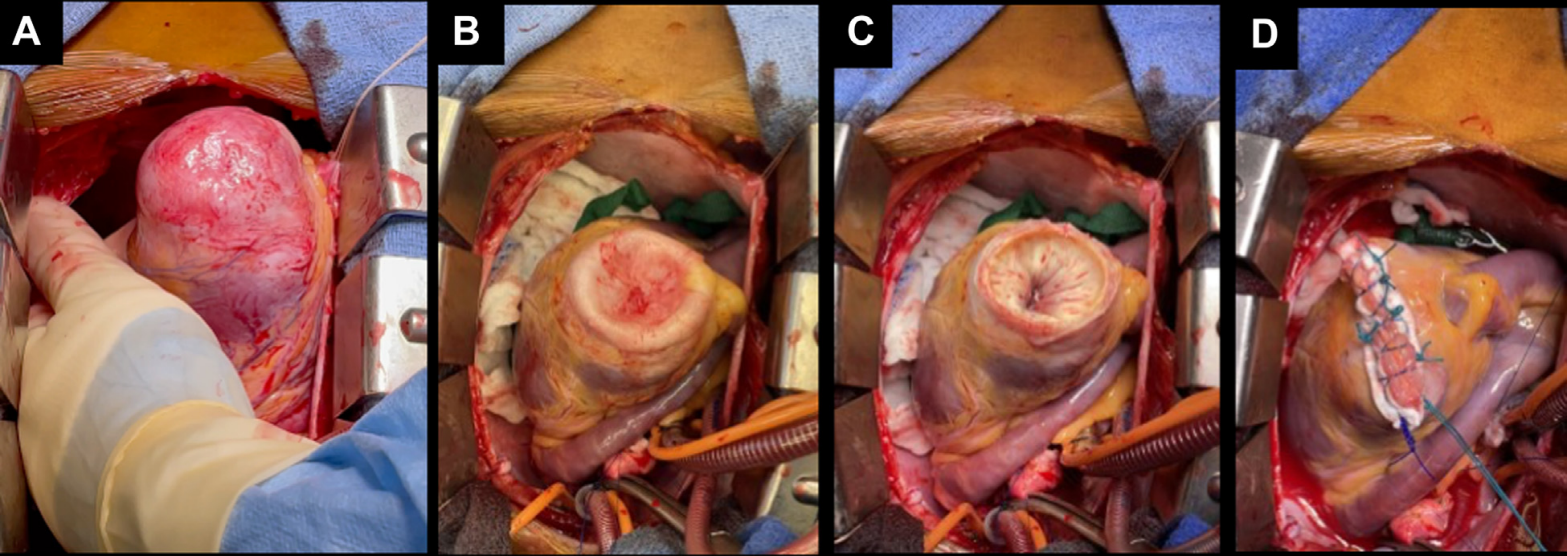
Aneurysmectomy Aneurysmectomy and myectomy performed for patient shown in Figures 1 and 2: (A) the protruding aneurysm when lifted out of the surgical field. See Video 2; (B) after venting the LV; (C) after resecting the aneurysm; (D) closure of the aneurysmectomy. Results of myectomy and aneurysmectomy are shown in Video 3. LV = left ventricular.

Nineteen patients (18%) had surgery at ages 56.6 ± 13 years. Eleven had surgery for NYHA functional class III symptoms, 8 had both symptoms and VT or VF, and 1 had refractory VT without symptoms. Of 8 patients with VT or VF, 7 had multiple previous ATPs, and 1 had VF. Table 5 shows operations that were performed and their outcomes. In every patient, direct surgical inspection showed fibrotic plaque on the mid-LV septum, anterior wall, and PMs corresponding to the site of mid-LV obstruction. Aortotomy and mid-LV resection from above were performed in 16 (84%) patients; ventriculotomy and mid-LV resection were performed from below in 13 (68%); resections were connected to provide unobstructed passage in 7 patients. Aneurysmectomy was performed in 12, and PM thinning or excision in 15. Aneurysmectomy was performed in patients with larger aneurysms, 2D echo volume (8.1 ± 7.9 vs 3.8 ± 5.7 ml, *P* < 0.01). Figure 9 and Video 2 show surgical images from the same patient as Figure 1, who had an aneurysmectomy, myectomy, and PM thinning. Video 3 shows the echocardiographic postoperative result. Two patients required repeat operations because of recurrence of mid-LV obstruction and limiting symptoms 2 and 6 years after their initial operation. The initial myectomy had been very extensive, but severe hyperkinesia recurred, not myocardial regrowth. In 1, persistent PM hypertrophy (left at the time of initial surgery) contributed to mid-LV obstruction; PMs were thinned at the second procedure to good effect. One patient who presented with collapse, hypotension, low cardiac output, and a large fibrotic aneurysm died in the postoperative period from hypotension, low cardiac output, and bowel infarction. The remaining 18 patients are alive 5.7 (IQR: 3.6-7.1) years after surgery. NYHA functional class was 3.1 ± 0.2 preoperative and 2.0 ± 0.7 postoperative (*P* < 0.001). Two patients were still NYHA functional class III. After surgery, in 8 patients with VT, it did not recur in 4, was greatly diminished in frequency in 1, and recurred in 3, requiring ATP. One patient operated on for symptoms developed an episode of VT only after surgery, treated with ATP.

**Table 5:** Surgical Indications, Procedures, and Outcomes in 19 Patients

Year of Surgery	Indication	Age at Surgery, y	Aortotomy	Ventricu-lotomy	Aneurysm-ectomy	PM Thinning or Excision	Functional Class Preoperative NYHA	Postoperative NYHA	Postoperative Course
2010	S and >20 VT, Rx'd ATP	56	X			X	III^b^	II	VT decreased in frequency and then no VT
2011	S and >20 VT, Rx'd ATP	57		X	X		III	II	
2014	S	64	X				III	II	
2014	S, low output, collapse	69		X	X		4	N/A	Postoperative hypotension low output and bowel infarct, died
2015	S	60	X			X	III	I	
2015	S and VF	45	X				III^b^	III	No recurrent VF or VT but recurrent symptoms after 2 y requiring reoperation
2017^a^	Recurrent S	47		X	X	X	III	II	Persistant symptoms; disopyramide with resolution
2015	S	64		X		X	III	III	Recurrent symptoms after 6 years requiring reoperation
2021^a^	Recurrent S	70	X	X	X	X	III	II	
2016	S	62	X				III	I	
2016	S and 4 VT ATP resistant to ablation	68	X	X	X	X	III^b^	II	VT after surgery Rx'd ATP
2016	S	30	X			X	III	III	
2017	S	69	X	X	X	X	III	II	
2018	S	45	X			X	III	I	VT only after surgery
2018	S	67		X	X	X	III	II	
2019	S	49	X	X	X	X	III	II	
2019	S and 3 VT ATP	58.00	X	X	X	X	III^b^	I	No VT
2019	VT storm >100 discharges refractory to VT ablation	70	X	X	X	X	III^b^	II	After 3 quiescent years; recurrent VT storm Rx'd VT ablation
2019	S and 2 VT ATP	53	X	X	X		III^b^	III	No VT
2021	>5 VT ATP	22	X	X	X	X	I^b^	I	No VT
2022	S and VT ATP	63	X			X	II^b^	I	On mexilitine and amio no VT after surgery; when amio stopped VT recurred

ATP = antitachycardia pacing; ICD = implanted cardioverter defibrillator; NYHA = New York Heart Association class; PM = papillary muscle; Rx'd = treated; S = symptoms; VF = ventricular fibrillation; VT = ventricular tachycardia.

a Surgery repeated for symptoms.

b ICD discharges.

## Discussion

This multicenter study evaluated the clinical course and treatment of HCM patients with apical aneurysm, focusing on the most pressing clinical issues. We found that the incidence of lethal or potentially lethal arrhythmias was associated with the presence of major HCM risk factors and larger aneurysm size. We found a high prevalence of clinical AF, 45%, with associated strokes; strokes without conventional cause were quite uncommon and of comparable incidence to severe bleeding risk. Myectomy surgery with aneurysmectomy when appropriate can provide improvement in heart failure symptoms (Central Illustration).

**Central Illustration:**
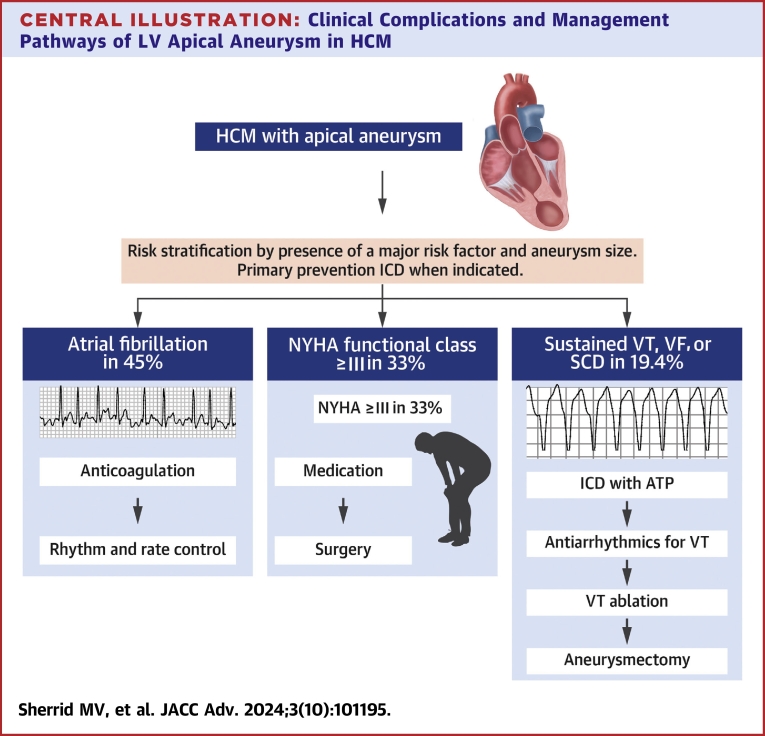
**Clinical Complications and Management Pathways of LV Apical Aneurysm in HCM** HCM = hypertrophic cardiomyopathy; LV = left ventricular.

We and others have previously shown that the overwhelming majority of HCM aneurysm patients (95%) have associated obstruction, defined as mid-LV complete systolic emptying.^3^^,^^5^ Aneurysms from obstruction often occur in the absence of high LV velocities because, when complete systolic emptying occurs, mid-LV flow is completely interrupted or greatly diminished.^5^ We have presented 7 lines of evidence to support the obstruction concept, including prior publications showing progression from mid-LV obstruction to aneurysm in 9% to 20% of patients.^5^ The primary underlying mechanism is supply-demand ischemia. Narrowed compressed intramural coronaries with impaired apical perfusion, ubiquitous in apical HCM ^9^ adversely affect supply, while high intra-aneurysm pressures due to obstruction adversely increase demand.

We confirmed the observation that patients with apical aneurysms have a high risk of ICD-aborted lethal or potentially lethal ventricular arrhythmias. Because of such prior observations, 56% of patients in this series had ICDs implanted during their course. Twenty-one patients (19.4%) had at least 1 appropriate ICD discharge or SCD, 16 (15%) had multiple ICD discharges, and 6 had VT storm. One patient was found dead. Only 2 patients had VF. Thus, the overwhelming majority of discharges were for VT treated with ATP. Overall, 33% of patients implanted had appropriate discharges, validating the primary prevention ICD strategy for selected patients. The question is which patients to implant: American and European guidelines differ.

### Clinical risk factors and subsequent VT, VF, or SCD

Of patients initially with a major risk factor, 55% subsequently had VT, VF, or SCD, compared with those without a risk factor in whom 10% subsequently had events. Of patients without prior sustained VT or cardiac arrest, among those who initially had a risk factor, 50% subsequently had VT, VF, or SCD, compared to 9% of those who did not. Thus, presence of a major risk factor at initial visit was a potent predictor of subsequent malignant arrhythmia. In contrast, calculated ESC risk score was much less sensitive with ≥6% score (=ICD should be considered) in only 16% of patients who had subsequent malignant arrhythmia.

### Aneurysm size

Patients with larger aneurysms were more likely to have appropriate ICD discharges or SCD. Patients with lethal or potentially lethal events had 2.5× greater biplane echo volumes than those who did not, 2× greater aneurysm area, and a 2.7× greater area on CMR. Patients with echo aneurysm area >4 cm^2^ had a quite high cumulative 5-year event rate of 35%, and those with echo aneurysm area >2 cm^2^ had a 5-year event rate of 17%, also above the usual threshold for consideration of an ICD. In contrast, patients with echo aneurysm area ≤2 cm^2^ had a low cumulative 5-year event rate of 6%, and those also without risk factors had an even lower rate. The present findings support the observations of Lee et al that patients with small aneurysms have very low event rates.^4^ Others found that the preponderance of patients with events had medium or large aneurysms.^1^ Shared decision-making with patients about ICD implantation is recommended. In our patients with the smallest aneurysm <2 cm^2^ and also without risk factors, VT, VF, or SCD occurred in only 2.5% at 5 years (Figure 7). Since in the great majority of cases apical aneurysms present as a *fait accompli* and do not enlarge over time, the data suggest it is reasonable to forgo ICD implantation in patients with the smallest aneurysms unless there is another compelling indication such as a major AHA/ACC risk factor. Periodic follow-up imaging and arrhythmia monitoring are appropriate.

We found no difference in LGE% in patients with and without ICD discharges, similar to others.^4^ LGE quantification is prone to limitations that may be phenotype-specific in apical aneurysms. We posit that thinning of the apical wall due to infarction and a limited number of tomographic slices at the apex both act to decrease the numerator in the mass LGE/mass LV formula. Instead of a quantitative LGE assessment, we found a suggestive trend qualitatively that patients with LGE just localized at the apical cap less frequently had malignant arrhythmia compared to those with dense LGE extending toward the level of obstruction.

### Atrial fibrillation and stroke

Clinical AF requiring anticoagulation was very common in our cohort, occurring in 49 (45.4%) patients; this was the indication for systemic anticoagulation in 47, while an additional 5 had anticoagulation because of thrombus in the aneurysm.

Others have found an incidence of stroke or embolism in aneurysm patients ranging from 1.1% to 2.9%/year.^1^^,^^4^ However, stroke can have diverse other causes, aside from embolization from an apical aneurysm. The high incidence of AF in the present series prompted us to evaluate whether strokes might have other origins besides the aneurysms. We adjudicated events as strokes according to recommendations of the AHA/ASA.^7^ The presence of a thrombus in an apical aneurysm was neither tabulated as an embolic event nor were transient ischemic attacks (TIAs) without imaging evidence of central nervous system damage. There are too many mimickers of TIA. Of 13 strokes that occurred in this cohort, 6 (46%) were attributed to AF either at its onset or during an anticoagulation hiatus. Two patients had occlusions of large extracardiac vessels, attributed in 1 to severe atherosclerotic stenosis and in the other to vertebral dissection. Five patients (4.6% of patients, 38% of the events) had strokes that had no explanation. Three occurred after our initial consultation with a low average incidence of 0.5% per year. Of note, none of 7 patients who had aneurysm thrombi had strokes, and none of 13 patients who had strokes had aneurysm thrombi. The unexplained stroke prevalence of 4.6% of our entire cohort must be contrasted with the risk of systemic anticoagulation and the prevalence of severe bleeding in 5 patients (4.6%), 4 while on anticoagulation. Bleeding risk should temper enthusiasm for anticoagulation in patients who do not have AF.

### Surgical myectomy and aneurysmectomy

Surgery for apical aneurysm in HCM is a challenging intervention that would not be recommended for a surgeon without substantial prior experience with extended myectomy. From direct surgical observation, a contact lesion of mid-LV subendocardial fibrosis in all of our patients supports the conclusion that obstruction is a central contributor to aneurysm formation. Surgical patients had NYHA functional class III symptoms; nearly half had also received appropriate discharges for lethal or potentially lethal ventricular arrhythmias (5 with multiple discharges). Surgical results must be evaluated in the context that these patients have exhausted medical and device therapy for symptoms.^8^ All patients had extended myectomy to relieve mid-LV obstruction with access either from above in 16, or below in 13, and both approaches connected in 7. The mid-LV walls contract circumferentially around hypertrophied central PMs, which act as a central plug focally contributing to obstruction.^5^ Consequently, 13 (72%) of patients had PM thinning. One patient who was in a low output state with an extremely large aneurysm died postoperatively; the rest are alive median 5.7 years later. Limiting symptoms improved after surgery by an average of 1 NYHA functional class. Two patients required reoperation 2 and 6 years after their initial surgery. Though symptomatic improvement is noted, surgery must be considered palliative given that patients still averaged NYHA functional class II afterward. Ventricular tachycardia was abolished in 4 patients, greatly decreased in 1 and recurred in 3.

### Prior reports

Our data confirm the prior report of Lee et al of an association between aneurysm size and the combined endpoint of appropriate ICD discharge and sudden death and, importantly, low risk for small aneurysm patients.^4^ We found that 38% of appropriate discharges or SCD occurred in patients >60 years of age, in agreement with Rowin’s observation; this suggests that for older patients with a major risk factor or a larger aneurysm, ICD implantation warrants serious consideration and that the “protection” of age in other HCM phenotypes does not apply to aneurysm patients.^10^

A recent thought-provoking editorial argued that LV aneurysms should not serve as an independent risk for ICD^11^, because monomorphic VT is the predominant arrhythmia and might not be lethal. Our data support the contention that monomorphic VT is the overwhelmingly predominant arrhythmia in these patients, not VF. However, 13 of 20 patients had VT treated with ATP on more than 1 day, and 6 had VT storm. Adding these to our 3 patients with VF or SCD, 43% of those with malignant arrhythmias had either VT storm, VF, or SCD. Moreover, sustained VT is often highly symptomatic and disruptive, especially when it is recurrent, and often results in hospitalization.^1^ Reliable treatment of VT is essential to allow a full lifestyle that might allow driving and climbing stairs. In the modern era, patients with anatomic heart disease with sustained or long runs of recurrent VT are treated with an ICD with ATP, not by observation or solely with antiarrhythmic drugs.

We found a very high incidence of clinical AF in our aneurysm patients (45.4%), either before their first visit or on follow-up. The prevalence of AF in aneurysm patients was 31% in 1 investigation and not tabulated in another.^1^^,^^4^

Rowin found a thromboembolic event incidence of 1.1%/year that did not differ from HCM patients without aneurysms.^1^ Lee found a thromboembolic rate of 2.9%/year.^4^ However, that latter research included patients with TIA as equivalent to stroke and also LV thrombus. We adjudicated strokes that occurred in our cohort according to AHA/ASA recommendations and did not include either TIA or LV thrombus. We found that the incidence of strokes, unexplained by either concomitant unanticoagulated AF or a large extracranial vessel cause, was low, 0.5%/year. In patients found to have LV thrombus in their aneurysms, we currently recommend anticoagulation, but it is not proven that these thrombi embolize. We currently do not routinely anticoagulate patients with aneurysms who have neither AF nor apical thrombus, and the low stroke rate we report reflects that practice. We acknowledge that nearly half the patients in our series were anticoagulated for AF and that this may have decreased cerebrovascular accident incidence.

### Limitations and proposals for further study

This was a retrospective study subject to their inherent limitations. Further study should confirm that patients with small aneurysms are at low enough risk to forego ICD implantation. High prevalence of AF should be confirmed. Stroke cause should be carefully adjudicated in future studies. Implanted arrhythmia monitoring to detect sustained VT or AF might logically have a role in aneurysm patients. Preventing arrhythmia recurrence without antiarrhythmic medication remains an unresolved problem. Since aneurysm formation is highly associated with mid-LV obstruction, myosin inhibition may play a future preventive role.

## Conclusions

Presence of a major risk factor and aneurysm size were highly associated with subsequent VT, VF, or SCD. The data support a primary prevention ICD in patients with 1 or more clinical risk factors or in larger aneurysms. Though HCM risk factors may prove to be more dispositive than aneurysm size there are often ambiguities about risk factors: eg, whether a SCD outside the circle of first-degree relatives should count, or ambiguity about their cause of death, or unexplained vs explained syncope. When such ambiguities exist, larger aneurysm size assumes a greater role in this judgement. From the data, it is a reasonable judgment to forgo ICD implantation in patients with the smallest aneurysms unless there is another compelling indication such as a major AHA/ACC risk factor. Clinically apparent AF is common in aneurysm patients occurring in 45%. Pending further study, we currently do not routinely anticoagulate patients with aneurysms who have neither AF nor apical thrombus. VT ablation and surgery reduced the frequency of symptomatic ventricular tachycardia; however, a sizable minority of patients experienced recurrent episodes requiring ATP and change of pharmacotherapy. Surgery though technically demanding can be effective in reducing heart failure symptoms resistant to medical therapy.

PERSPECTIVES **COMPETENCY IN PATIENT CARE:** The data support the use of primary prevention ICD in patients with 1 or more clinical risk factors and in larger aneurysms. In patients with both small aneurysm area ≤2 cm^2^ and no major risk factors, it is reasonable to forego ICD implantation. Imaging follow-up is appropriate, though change in aneurysm size is uncommon. Prevalence of clinical AF was high, 45% and anticoagulation should be started promptly for clinically apparent AF. Incidence of stroke without conventional risk factors such as atrial fibrillation and large vessel cerebral occlusive disease was low, 0.5%/year. Surgery, performed in 19% of our patients, was effective in reducing symptoms that had been resistant to medical therapy.

**TRANSITIONAL OUTLOOK:** Implanted arrhythmia monitoring might prove clinically useful. More complete prevention of arrhythmia recurrence is an unresolved problem. Myosin inhibition might prevent progression of mid-LV obstruction to aneurysm.

## Funding support and author disclosures

The authors have reported that they have no relationships relevant to the contents of this paper to disclose.

## Supplementary Data

Supplemental material

Video 1 LV Cineangiogram of Patient Shown in Figure 1.

Video 2 Heart lifted out of surgical field to demonstrate the aneurysm.

Video 3 Postoperative echocardiograms.
