# Supplementary material for: Clinical Course and Treatment of Patients With Apical Aneurysms Due to Hypertrophic Cardiomyopathy
Source: JACC Adv. 2024 Aug 27;3(10):101195. doi: 10.1016/j.jacadv.2024.101195 (PMC11400613; doi:10.1016/j.jacadv.2024.101195)
Supplement: Supplemental material [file mmc1.docx]

**Supplemental Appendix**

**Supplemental methods:**

**American major risk factors for recommendation of ICD Placement in high-risk patients with HCM (excluding apical aneurysm)**

For adult patients with HCM with ≥1 major risk factors for SCD, it is reasonable to offer

an ICD. These major risk factors include:

a. Sudden death judged definitively or likely attributable to HCM in ≥1 first-degree or

close relatives who are ≤50 years of age;

b. Massive LVH ≥30 mm in any LV segment;

c. ≥1 Recent episodes of syncope suspected by clinical history to be arrhythmic (ie, unlikely to be of neurocardiogenic [vasovagal] etiology, or related to LVOTO);

d. LV systolic dysfunction (EF <50%).

**Major bleeding (as defined by the International Society on Thrombosis and Haemostasis)**

- Fatal bleeding, and/or

- Symptomatic bleeding in a critical area or organ, such as intracranial, intraspinal, intraocular, retroperitoneal, intra-articular or pericardial, or intramuscular with compartment syndrome, and/or

- Bleeding causing a fall in hemoglobin level of 2 g/dl (1.24 mmol/dl) or more, or leading to transfusion of two or more units of whole blood or red cells.

**Echocardiography.** All echocardiographic measurements were performed de novo with separate rigorously defined, pre-specified research quantification on dedicated workstations. Echo aneurysm size and wall thicknesses were only quantified in patients who received echo contrast or where entire endocardium was clearly seen and could be unequivocally traced. We measured area and volume of the aneurysm in 2 orthogonal apical views, tabulating the average. We measured maximal mid-LV wall thickness, and basal wall thicknesses.

**CMR:** CMR imaging was performed on Siemens, Phillips and, early in the study period, on GE CMR systems. Image acquisitions included balanced steady-state free precession cine images in 2-, 3- and 4-chamber long-axis planes and a stack of short-axis images from the mitral valve to the apex. LGE was acquired for fibrosis quantification with 2D segmented inversion-recovery gradient echo sequences in the same imaging planes, acquired 10-15 min after administration of gadolinium contrast.

Separate research reviews of all non-contrast images were performed with pre-specified measurement criteria. Aneurysm area was measured in 2 orthogonal long-axis views, tabulating the average. We measured maximal diastolic mid-LV wall and basal anterior septal thickness. Percent of LV mass with LGE was determined, as described previously by Chan et al, on dedicated workstations with image plane co-localization capabilities. The value obtained from the clinical report was tabulated.

**Supplemental Results and Discussion**

Racial distribution was White n=63 (58.3%), Black n=27 (25.0%), Asian n=14 (13.0%) and Hispanic n=4 (3.7%). Compared to the NYU HCM Registry, patients with aneurysms from the 3 centers more commonly were Black or Asian, 38% vs 19% (p<0.0001) and patients from the NYU aneurysm cohort alone were also more commonly Black or Asian, 43% vs 19% (p<0.0008).

We previously observed that aneurysms were common in Black patients in our cohort at NYU. We now confirm that Black and Asian patients comprise 40% of HCM aneurysm patients, a racial frequency much more common than we observed in our overall NYU HCM database (19%) and more frequent than the racial frequency in HCM databases reported by others.

**Supplemental references:**

Levine GN, McEvoy JW, Fang JC et al. Management of Patients at Risk for and With Left Ventricular Thrombus: A Scientific Statement From the American Heart Association. Circulation 2022;146:e205-e223.

Arabadjian ME, Yu G, Sherrid MV, Dickson VV. Disease Expression and Outcomes in Black and White Adults With Hypertrophic Cardiomyopathy. J Am Heart Assoc 2021;10:e019978.

Arabadjian M, McCarthy M, Dickson VV. An Integrated Review of Hypertrophic Cardiomyopathy in Black Populations: Underrecognized and Understudied. J Cardiovasc Nurs 2021;36:104-115. <http://dx.doi.org/10.1016/j.ijcard.2014.04.103>

Papanastasiou CA, Zegkos T, Karamitsos TD et al Prognostic role of left ventricular apical aneurysm in hypertrophic cardiomyopathy: A systematic review and meta-analysis. Int J Cardiol 2021; 127-132. doi: 10.1016/j.ijcard.2021.03.056

Chan RH, Maron BJ, Olivotto I et al. Prognostic value of quantitative contrast-enhanced cardiovascular magnetic resonance for the evaluation of sudden death risk in patients with hypertrophic cardiomyopathy. Circulation 2014;130:484-95.

Lim K-K, Maron BJ, Knight BP. Successful catheter ablation of hemodynamically

unstable monomorphic ventricular tachycardia in a patient with

hypertrophic cardiomyopathy and apical aneurysm. J Cardiovasc Electrophysiol.

2009;20:445–7.

Dukkipati SR, d’Avila A, Soejima K, et al. Long-term outcomes of combined

epicardial and endocardial ablation of monomorphic ventricular

tachycardia related to hypertrophic cardiomyopathy. Circ Arrhythm Electrophysiol.

2011;4:185–94.

Strachinaru M, Huurman R, Bowen DJ, Schinkel AFL, Hirsch A, Michels M. Relation between

early diastolic mid-ventricular flow and elastic forces indicating aneurysm formation in

hypertrophic cardiomyopathy. J Am Soc Echocardiogr. 2022;35:846–856.e2.

Minami Y, Kajimoto K, Terajima Y, et al. Clinical implications of midventricular obstruction in patients

with hypertrophic cardiomyopathy. J Am Coll Cardiol. 2011;57:2346–2355.

Minami Y, Haruki S, Hagiwara N. Phenotypic overlap in hypertrophic cardiomyopathy: apical

hypertrophy, midventricular obstruction, and apical aneurysm. J Cardiol. 2014;64:463–469

Batzner A, Kraus N, Reiter T, Morbach C, Seggewiss H. Development of apical aneurysm in

apical HCM. Eur Heart J Cardiovasc Imaging. 2021;22:e74.
